# Effects of *Enterobacter cloacae* HG-1 on the Nitrogen-Fixing Community Structure of Wheat Rhizosphere Soil and on Salt Tolerance

**DOI:** 10.3389/fpls.2020.01094

**Published:** 2020-07-17

**Authors:** Chao Ji, Zhaoyang Liu, Liping Hao, Xin Song, Changdong Wang, Yue Liu, Huying Li, Chaohui Li, Qixiong Gao, Xunli Liu

**Affiliations:** ^1^ College of Forestry, Shandong Agriculture University, Taian, China; ^2^ State Forestry and Grassland Administration Key Laboratory of Silviculture in Downstream Areas of the Yellow River, Taian, China; ^3^ College of Plant Conservation, Shandong Agriculture University, Taian, China

**Keywords:** salt stress, nitrogen-fixing bacteria, plant growth promotion, community structure, high-throughput sequencing

## Abstract

The present study investigated the physiological and biochemical characteristics of *Enterobacter cloacae* HG-1 isolated from saline-alkali soil. We further studied the effect of this strain on the salt tolerance of wheat and on the community structure of nitrogen-fixing bacteria in rhizosphere soil. We determined that the investigated strain had high nitrogen fixation activity and produced iron carriers, 1-aminocyclopropane-1-carboxylic acid deaminase, and plant hormones. The metabolites of this strain contained 2,3-butanediol, [R-(R*, R*)], 2-heptanone, and other growth-promoting and antibacterial substances. The strain was also highly salt-tolerant (10% NaCl). After the inoculation of wheat with the HG-1 strain, we recorded increases in root length, plant height, fresh weight, and dry weight of 19.15%, 18.83%, 16.67%, and 17.96%, respectively, compared with uninoculated plants (*P* < 0.05). Compared with the leaves of uninoculated plants, the proline concentration in the leaves of inoculated plants increased by 12.43% (*P* < 0.05), the malondialdehyde level decreased by 27.26% (*P* < 0.05), K^+^ increased by 20.69%, Ca^2+^ increased by 57.53% and Na^+^ decreased by 31.43% (all *P*<0.05). Furthermore, we detected that inoculation with the HG-1 strain did not affect the species composition of nitrogen-fixing bacteria in wheat rhizosphere soil at the phylum level. However, the average relative abundance of Proteobacteria was significantly increased, whereas the abundance of Verrucomiorobia was significantly decreased compared with uninoculated plants. At the genus level, we detected 32 genera in control samples and 27 genera in inoculated samples, and the species diversity and relative abundance of samples inoculated with the HG-1 strain decreased compared with uninoculated plants. Inoculated samples had lower abundances of *Azospirillum*, *Rhodomicrobium*, and *Anabaena*. Our study demonstrated that the inoculation of wheat with *E. cloacae* HG-1 could promote the growth of wheat under salt stress and increase salt stress tolerance. The results of this study investigating the interaction among soil, plants, and microorganisms supplement agricultural microbial databases and could provide a reference for the development of microbial-based saline soil improvement programs.

## Introduction

Salinity is one of the major abiotic stresses, with a total of 3% of the world’s land mass being affected by salinity (Mukhtar et al., 2018). Global increase in soil salinization constitutes a most devastating environmental threat for crop yield and food quality (Asrar et al., 2017). In China, nearly 37 million hectares of saline-alkali soil, accounting for 4.9% of the nation’s arable land (Brandon and Ramankutty, 1994). However, it also remains seemingly unavoidable in the short- to middle-term that global food demand will rise as a result of population growth and environmental degradation (Ervin and Lopez-Carr, 2018). Furthermore, the effects of climate change, such as decreased rainfall and high temperatures, will further increase the degree of salinization (Shrivastava and Kumar, 2015), which will induce an estimated 70% reduction in the cultivation of wheat and other major crops (Acquaah, 2007). The transformation of salt-tolerant crops does not have a high success rate because of the complex mechanism of salt stress in crops (Dodd and Pérez-Alfocea, 2012; Kumari A. et al., 2015) despite considerable efforts in traditional breeding and genetic engineering (Dodd and Pérez-Alfocea, 2012; Joshi et al., 2015; Krishna et al., 2015). However, one study reported that plant growth–promoting (PGP) rhizobacteria (PGPR) significantly promoted the growth of plants under salt stress, which led to an increase in research surrounding PGPR mechanisms and the structure of plant rhizosphere microbial communities (Qin et al., 2016).

PGPR can establish a symbiotic relationship with plants and promote their growth under both normal and stress conditions (Desgarennes et al., 2015). Symbiotic bacteria exist in all plants, and this relationship may be a key factor involved in plant stress tolerance. In fact, local adaptation of plants to their environment is driven by the genetic differentiation among closely associated PGPRs (Rodriguez et al., 2008). Transplanting various plant species in the absence of bacteria is notoriously difficult (Leifert et al., 1989), and this difficulty supports the importance of bacteria to plant growth, including under stressful conditions (Singh and Jha, 2016). The rational use of PGPR is a valuable approach for reducing salt stress in plants (Bisseling et al., 2009). These bacteria exhibit multiple stress-related traits that may contribute to their plant protective capabilities under growth inhibiting levels of salt (Rohban et al., 2009; Siddikee et al., 2010; Bharti et al., 2013; Sharma et al., 2016). In naturally salt-affected soil, PGPR without salt tolerance characteristics gradually lose their plant growth-promoting (PGP) characteristics with an increase in salinity (Upadhyay et al., 2009; Etesami and Beattie, 2018). The development and utilization of salt-tolerant PGPR are feasible measures for increasing crop yield under salt stress (Sáenz-Mata et al., 2016). PGPR are involved in various biological processes such as the mobilization of soluble phosphorus (Zhu et al., 2011); fixation of nitrogen (Yan et al., 2015); increase of antioxidant enzymes levels (Ullah and Bano, 2015); regulation of ion transport protein expression (Etesami, 2018); secretion of extracellular polysaccharides (Qin et al., 2016); and production of plant hormones, iron carriers, and ACC deaminases (Ji et al., 2020). PGPR can also alter the root structure, morphology, hydraulic conductivity, and hormonal status and can release various volatile compounds (such as glutamic acid, proline, and peptide) associated with stress accumulation infiltration, killing pathogens (Arora et al., 2013; Olanrewaju et al., 2017). These mechanisms are related to improvements in plant salt tolerance and an induced system tolerance (IST) (Kaushal and Wani, 2016; Etesami and Beattie, 2018). Rhizosphere soil is a dynamic and complex element in farmland ecosystems (Yang et al., 2016). Soil microorganisms are important drivers of plant diversity and productivity in terrestrial ecosystems (Van et al., 2008). It is roughly estimated that about 25% of plants are dependent on nitrogen-fixing bacteria in natural soils with poor nutrients (Van et al., 2006).

The diversity, relative abundance, and activity of microbial communities play a central role in soil organic matter decomposition, nutrient recycling, system stability, and anti-interference ability (Cardinale et al., 2006; Céline et al., 2007; Enwall et al., 2007; Quadros et al., 2012). However, salinity has a strong filtering effect on bacterial community, and the change of community salt tolerance is partly driven by the change of community composition (Rath et al., 2018). When inoculated with salt tolerant microorganisms, it is likely to cause the recovery of respiration and growth function of microbial community in the soil (Rath et al., 2019). The identification of plant-related bacteria, analysis of their interactions with their host, and knowledge regarding manners in which these interactions promote the survival of both organisms are critical in developing strategies for the protection of these plants (Ruppel et al., 2013).

The nitrogen cycle is one of the main nutrient cycles in terrestrial ecosystems. This nutrient cycle is a globally vital biogeochemical process mediated almost entirely by microorganisms in the environment (Wang et al., 2015b). Nitrogen fixation is performed by microorganisms in the natural nitrogen cycle (Levy-Booth et al., 2014). The chemical form and content of nitrogen play a central role in regulating the composition of the soil microbial community (Fierer et al., 2011; Giagnoni et al., 2015). Nitrogen-fixing bacteria (NFB) is a PGPR with the unique ability to fix N_2_ from the atmosphere into ammonium cations (NH_4_^+^), which are available for plant uptake (Alfaro-Espinoza and Ullrich, 2015). The global quantity of biological nitrogen fixation is estimated at approximately 2×10^13^ g N/year globally (Falkowski, 1997). Therefore, further understanding of the community structure of NFB in the crop rhizosphere soil would be valuable. All nitrogen-fixing microorganisms contain the *nifH* gene. The *nifH* gene encodes for the nitrogene-fixating protein ferritin, and the phylogeny of the *nifH* gene is consistent with 16S rRNA (Levy-Booth et al., 2014). Therefore, the *nifH* gene is an ideal target for studying nitrogen-fixing microorganisms (Raymond et al., 2004). High-throughput sequencing technology can be used to compare the diversity and relative abundance of microbial species among different samples in order to investigate mechanisms underlying microorganisms soil plant interactions (Delong, 2002). Despite an increase in the use of microbial inoculants and numerous studies investigating PGPR, the effects of microbial inoculants on nitrogen-fixing microbial communities in the crop rhizosphere soil under salt stress are poorly understood.

Wheat is one of the most important cereals worldwide for human diet. Although wheat is extensively cultivated in the area of Yellow River Delta, its area of cultivation is limited due the excess of salinity and alkalinity of soils (Liu et al., 2017). The “Bohai sea granary programme” that was recently launched in China has for objective to better exploit the salt-alkaline land for cereal cultivation. Therefore, the purpose of the present study was (i) screen strains with high salt tolerance and prominent nitrogen-fixing and other life-promoting characteristics from saline alkali soil of the Yellow River Delta, (ii) to test their effects on wheat growth under salt stress, and (iii) to analyze the effects of salt stress on the community structure of NFB in wheat rhizosphere soil. These results may provide new data and insights into future screening of PGPR in the rhizosphere of plants under salt stress.

## Materials and Methods

### Isolation and Characterization of the Strain

In this study, the soil used to isolate strains was collected from wheat rhizosphere soil in the secondary salinization area of the Yellow River Delta, Shandong Province of China (118°49ʹ15″E, 37°24ʹ31″N). Ten grams of soil was mixed in 100 ml of sterile water and serially diluted to 10^-4^, plated on NA plates (8% NaCl), and incubated at 30°C ± 2°C for 3 to 4 days. The obtained salt-tolerant strains were inoculated into a nitrogen-free JNFb agar plate and continuously cultured at 30°C for 5 days to observe the bacterial growth as the evidence of nitrogen-fixing activity (Döbereiner, 1995).

The bacterium was subcultured twice. Finally, the isolates were streaked onto NA medium. A glycerol stock solution (30% v/v) of the isolate was prepared and stored at –80°C for later use. Based on colony morphology differences, 10 isolates were identified. Next, we tested the nitrogen fixation activity of the isolates (Marciano et al., 2012) and selected the strain with the highest nitrogen fixation activity for pot experiment.

Primary characterization of the test organism was done by fundamental microbiological and biochemical tests including Gram staining, Voges-Proskauer test, citrate utilization test, oxidase, aerobism, starch hydrolysis, catalase, and nitrate reductase following standard methods (Prescott and Harley, 1999). Using the BIOLOG Microstation™ system (BIOLOG Inc., Hayward, CA), the GEN III orifice 96 was tested to confirm the carbon source utilization and chemical sensitivity of bacteria to Rifamycin SV, Minocycline, etc.

### Amplification and Sequencing of 16S rRNA Genes

To identify the bacteria at the molecular level, the universal primers 27F (5′-AGAGTTTGATCMTGGCTCAG-3′) and 1492R (5′-TACGGYTACCTTGTTACGACT-3′) were used to amplify the 16S rRNA bacterial gene sequences *via* PCR (Lane, 1991). The 16S rRNA sequences obtained from the strains by PCR were compared to those from the NCBI database. Pairwise evolutionary distances between the 16S rRNA sequence of the HG-1 test isolate and its related bacterial strains were calculated. A phylogenetic tree was constructed by the Neighbor-Joining method using MEGA v.5.0 (Tamura et al., 2011). Neighbor-Joining and bootstrap analyses were performed with 1,000 bootstrap replications. The evolutionary distances were computed by the Maximum Likelihood method (Tamura et al., 2011). The 16S rRNA gene sequence of HG-1 was deposited in the NCBI database under the accession No. SUB6437962 Seq1 MN582993.

### Bioassays for the Promotion of Growth and Enhancement of Salinity Tolerance Traits

To investigate activity of phosphorus and potassium dissolution, we tested whether the bacterial colonies on NBRIP and silicate solid medium produce a clear circle, if formed, it is an indication of positive result showing a dissolved phosphorus and potassium (Mehta and Nautiyal, 2001). The dissolution efficiency of phosphorus and potassium in the strain was quantitatively analyzed by methods described in Bray and Kurtz (1945) and Lü and Huang (2010). The assay method was used to detect the nitrogenase activity of the strain (Berge et al., 2002). The Salkowski analysis was used to analyze the content of IAA produced by the strain in a liquid culture containing l-tryptophan (0.5 mg/ml) within 48 h (Moses et al., 2015). The activity of ACC deaminase was determined with the method described in Penrose and Glick (2003). Strain culture was inoculated on chrome azurole S (CAS) agar plate and incubated at 30°C for 4–5 days. After the experimental period, we observed if an orange formation would be visible around the bacterial colonies to test whether the strain produced iron carriers (Schywn and Nielands, 1987).

The activated strain sample was inoculated in 50 ml LB liquid medium, and cultured at 28°C for 14 to 16 h. The fermentation liquid was then extracted and inoculated into another medium (soluble starch 100 g/L, soybean cake powder 18 g/L, magnesium sulfate 0.75 g/L, potassium dihydrogen phosphate 1.0 g/L, pH 5.5) in a ratio of 2% (V/V) for 7 days. The fermentation liquid was then isolated after shaking the culture at 30°C, 200 r/min. The collected liquid was then centrifuged at 4°C and 10,000 r/min, for 2 min, to isolate and collect the supernatant. The C18 solid phase extraction column was initiated by adding 6 ml of methanol and 6 ml of 10% methanol, respectively, and the collected supernatant was slowly injected into the column at a rate of 0.8 ml/min. After the sample was added, it was rinsed twice with 6 ml of 10% methanol, and the effluent was discarded. Finally, it was eluted twice with 5 ml of 80% methanol and the eluate was collected. The eluate was air-dried at room temperature (23°C ± 2°C), dissolved in methanol and transferred to 2 ml centrifuge tube, and stored in a 4°C incubator. An Agilent ZORBAX Eclipse Plus C18 column (250 × 4.6 mm, 5 μm) was used. The mobile phase was a binary mixed solvent of methanol-water (containing 0.2% glacial acetic acid) in a volume ratio of 2:3. The column temperature was set to 35°C, with a flow rate of 0.8 ml/min, the injection volume was 10 μl, and the detection wavelength was 254 nm. Gibberellin Acid (GA_3_) and Zeatin (ZT) standards (purity > 98%) were purchased from Shanghai Aladdin Biochemical Technology Co., Ltd. The contents of GA_3_ and ZT were calculated from the standard curve.

The activated colonies were scribed into LB medium, cultured at 3°C for 24 h, and the samples were extracted at 40°C by PDMS/DVB65μm solid-phase microextraction head for 30 min. The extraction head was transferred to GCMS-TQ8050 (Shimadzu, Japan). Rtx-5MS capillary column (60 m × 0.25 µm ID × 0.25 µm thickness film) was used. The initial column temperature was maintained at 40°C, after 3 min, the temperature was increased to 160°C at the rate of 8°C/min, maintained for 2 min, and then increased to 240°C at the rate of 15°C/min, maintained for 3 min. Total operation time was 28.33 min. Helium was used as carrier gas and the flow rate is kept at 1.50 ml/min. The mass spectra of unknown compounds were compared with NIST17 and NIST17s (National institute of Standards and Technology) standard mass spectrometry libraries to determine the structure of the substance corresponding to the peaks. The ratio of peak area to total peak area is the relative content of each volatile component.

### Plant Materials and Treatments

Wheat cv jimai 21 seeds (provided by the College of Agriculture, Shandong Agricultural University) were rinsed with clean water, soaked in 75% ethanol for 10 min, then soaked in 30% sodium hypochlorite for 30 to 60 s, rinsed with sterile water five to six times, and dried. The soil used for potted plants was obtained at 0 to 20 cm of depth in wheat fields in the Yellow River delta (118°41′07′′E, 37°17′17′′N) (Dongying City, Shandong, China) in October 2018. A 2-mm sieve was used to remove rubble, roots, and other debris. We filled a clay pot with an inner diameter of 30 cm with 3.0 kg of soil per pot. We selected uniform wheat seeds and planted them in a pot, (10 grains per pot) watered them abundantly, and managed by conventional methods (Reddy and Sudhakara, 2014). When the wheat seedlings grew to about 5 cm, we choose 20 pots with plants with the same growth rate. Among them, ten pots were inoculated with HG-1 bacterial suspension. The bacterial inocula were prepared using sterile Milli-Q water by re-suspending cells (10^8^ CFU/ml) harvested from nutrient broth and a volume of 20 ml suspension was poured around the roots of the seedlings in each pot. Ten pots were treated with the same amount of sterile Milli-Q water (20 ml) and marked as the blank control group, CK (Wei et al., 2017). Randomized complete block design was used in this study (Wang et al., 2018). After 30 days of cultivation, wheat plants were taken out of the pots and plant biomass was measured. Ten soil cores were randomly selected from pots with different treatments and the rhizosphere soil was carefully collected, completely homogenized using a 2-mm sieve, and stored at −80°C for microbial community structure analysis (Wang et al., 2018).

### Soil Sampling and Analysis

Soil sample used for plant growth studies was analyzed for its various chemical properties. Soil and water were mixed in 1:2.5 ratio; pH and electrical conductivity (EC) was measured with pH meter and conductivity meter (Mettler Toledo, Switzerland). The organic carbon content in the soil was determined with a method described in Aj et al. (1934). Soil available phosphorus (Olsen P) was determined using the method proposed by Olsen et al. (1954). Available nitrogen and exchangeable potassium values were obtained by a method described in Jackson (1979). For ion analysis, 0.2 g of wheat rhizosphere soil was treated with 1 ml deionized water and 5 ml concentrated sulfuric acid overnight, and then the cooked liquid was fixed to 50 ml. Measurements were carried out on 1 ml of the solution, which was extracted and diluted 10 times. Na^+^, K^+^ and Ca^2+^ content was measured *via* inductively coupled plasma optical emission spectroscopy (ICP, Thermo Scientific™ iCAP™ 7000 Plus, USA) (Singh and Jha, 2016).

### Biomass and Antioxidant Activity of Wheat Plants

Plant growth was measured using root length, shoot length, fresh weight, and dry weight (Singh and Jha, 2016). Total soluble sugar content was determined using the method described by Irigoyen et al. (2006), and the proline content in wheat leaves was determined using the previously described method by Bates et al. (1973). Lipid peroxidation levels were determined by measuring malondialdehyde (MDA) from thiobarbital acid (TBA) reaction using the method described in Hodges et al. (1999). After the non-specific absorbance was removed, MDA concentration was determined according to its molar extinction coefficient (155 nm^−1^·cm^−1^), and the result was expressed as mmol·g^−1^ FW.

### Determination of Ion Content of Wheat Plants

According to the method described by Singh and Jha (2016), 1 g of bud tissue was acquired from plants of each treatment and mixed with a solution of perchloric acid, sulfuric acid, and distilled water in the ratio of 10:1:2. The contents of Na^+^, K^+^, and Ca^2+^ were determined using ICP. Each sample analysis was repeated three times to ensure its accuracy.

### PCR Amplification and Illumina MiSeq Sequencing

Three rhizosphere soil samples were combined into one DNA sample, and we prepared three replicate DNA samples per experiment (eighteen rhizosphere soil samples total). The soil samples were sequenced by the Majorbio company (China). Soil DNA Kit (Omega, Bio-Tek, USA) was used to extract total soil DNA. PCR was done using the extracted genomic DNA as a template, with *nifHF* (AAAGGYGGWATCGGYAARTCCACCAC) and *nifHR* (TTGTTSGCSGCRTACATSGCCATCAT) as primers. The PCR reaction system included: 5 × FastPfu Buffer (4 μl), 2.5 mM dNTPs (2 μl), 0.8 μl of each for forward and reverse primers (5 μM), FastPfu Polymerase (0.4 μl), BSA (0.2 μl), template DNA (10 ng), and ddH_2_O was added to 20 μl. The PCR procedure for the amplification of *nifH gene* was as follows: pre-denaturation at 95°C for 3 min, denaturation at 95°C for 30 s, annealing at 55°C for 30 s, 37 cycles of extension at 72°C for 45 s, and a final extension at 72°C for 10 min. Purified PCR products were quantified by Qubit^®^3.0 (Life Invitrogen) and every twenty-four amplicons whose adapters and barcodes were different were mixed equally. The pooled DNA product was used to construct Illumina Pair-End library following Illumina’s genomic DNA library preparation procedure. Then the amplicon library was paired-end sequenced (2 × 300) on an Illumina MiSeq platform (Shanghai BIOZERON Co., Ltd) according to the standard protocols.

### Processing of Illumina MiSeq Sequencing Data

The QIIME 1.17 software was used to analyze and screen the sequencing results. The sequences with poor sequencing quality were removed and the sequence length was screened. The sequences were determined to be the final sample sequences according to barcode. Data decontamination method and parameters were: 1. the bases were filtered with a mass value below 20 at the end of the read with a window of 50 bp. If the average mass value in the window was lower than 20, we cut off the back-end bases from the window and filtered the read below 50 bp after quality control; 2. According to overlap relation between PE reads, pairs of reads were merged into a sequence with the minimum overlap length of 10 bp; 3. The maximum allowed error matching ratio in the overlapping region of the splicing sequence was 0.2, and the non-conforming sequences were screened; 4. We differentiated the samples according to barcode and primer at both ends of the sequence and adjusted the direction of the sequence. The allowed barcode mismatches equaled 0 and the maximum primer mismatches equaled 2.

In the HG-1 group and the CK, MiSeq sequencing of *nifH* genes resulted in 109,119 and 152,540 high quality and chimera-free reads, respectively. The high-quality sequences were clustered into operational taxonomic unit (OTU) by the Usearch software (version 7.1). The non-repeating sequences (excluding single sequences) were clustered into OTUs according to 97% similarity, and the representative sequences of OTU were obtained by removing chimeras in the clustering process. The classification information of each OTU was obtained by annotating 97% similar OTU representative sequences with RDP classifier Bayesian algorithm. The Mothur software (version 1.30.1) was used to evaluate Chao, Ace, and Shannon indices at around 97%. Principal component analysis (PCA) based on Bray-Curtis distances was used to analyze the overall structural changes of NFB communities in rhizosphere soil of wheat inoculated and uninoculated with HG-1 strain. Species abundance of each sample was calculated at different taxonomic levels. The community composition of control and HG-1 species was presented in the community pie chart. The species whose abundance accounted for less than 0.001 in all samples were classified as “others.” All raw reads were deposited into the NCBI Sequence Read Archive (SRA) database (Accession Number: SUB6522639).

### Statistical Analysis

All experiments were performed in triplicates. The SPSS 19.0 software (IBM, Chicago, USA) was used for one-way ANOVA of parameters of plants and soil. Significance was calculated by Student’s t-test. Differences in mean values were considered significant when *P* < 0.05. Differences between NFB on the phylum level in CK and HG-1 samples were assessed using two-tailed Student’s t-tests.

## Results

### Isolation, Biochemical Characterization, and Identification of HG-1

**Figure 1:**
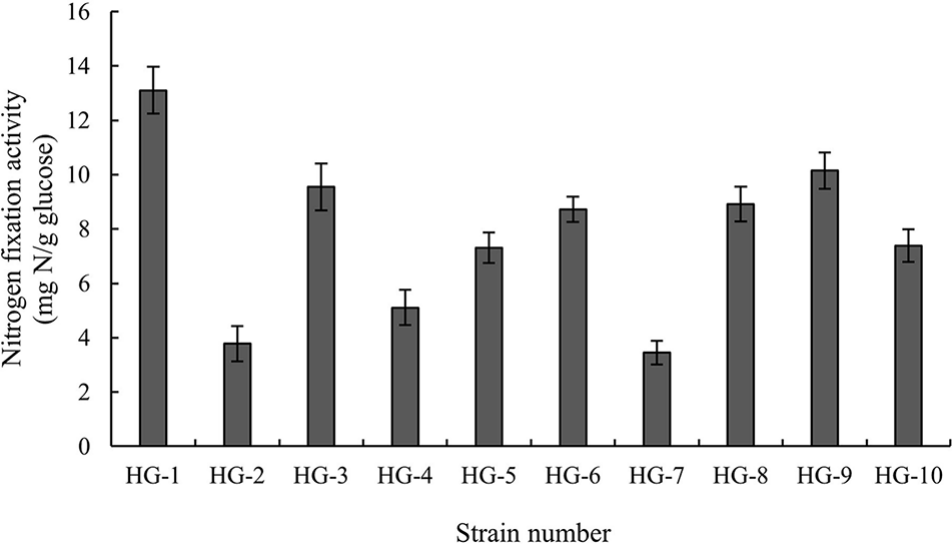
The results of nitrogen fixation activity determination of the isolates.

Based on colony morphology, 10 strains of bacteria capable of growing on nitrogen free JNFb agar plate were isolated from the soil sample of the Yellow River delta. Nine strains were gram-positive and HG-1 was gram-negative. The nitrogen-fixing activity of HG-1 strain was measured quantitatively, and the nitrogen-fixing activity of HG-1 strain was the highest (13.105 ± 0.858 mg N/g glucose) (**Figure 1**). The measurements of the physiological and biochemical indices of the isolated HG-1 strain revealed that the strain was a facultative anaerobe. The results of oxidase, starch hydrolysis, and urease tests were negative, whereas those of catalase, nitrate reduction, V-P, 5% NaCl, 10% NaCl, gelatin liquefaction, and citrate utilization tests were all positive. The isolated strain could utilize salicin, L-rhamnose, and inosine among other compounds. The complete list is presented in **Table 1**. Furthermore, the results of the antibiotic sensitivity analysis revealed that the HG-1 strain was resistant to rifamycin SV, troleandomycin, 1% sodium lactate, lincomycin, guanidine HCl, tetrazolium blue, tetrazolium violet, and vancomycin (**Table 1**). A 16S rDNA sequence alignment demonstrated that the strain belonged to the species *Enterobacter cloacae*. The phylogenetic tree constructed using MEGA software is presented in **Figure S1**. The salt-tolerant strains of saline soil were screened out using an 8% NaCl NA medium. The strains were then cultured in a JNFb-AGAR plate (no nitrogen source), silicate, or NBRIP media. We found that *Enterobacter cloacae* HG-1 grow normally in JNFb-AGAR plate (no nitrogen source). We further detected that the HG-1 strain formed transparent areas in both NBRIP and silicate media.

**Table 1:** Biochemical and physiological characteristics of HG-1.

Characteristic	Result	Carbohydrate	Utilization	Carbohydrate	Utilization	Chemical Sensitivity Assays	Reaction
Grams stain	−	Dextrin	○	Salicin	+	Rifamycin SV	+
Oxidase	−	d-Maltose	+	N-Acetyl-D-Glucosamine	+	Minocycline	−
Aerobism test	Facultative anaerobic	d-Trehalose	+	N-Acetyl-β-D-Mannosamine	+	D-Serine	○
Catalase test	+	d-Cellobiose	+	N-Acetyl-D-Galactosamine	+	Troleandomycin	+
Nitrate reductase	+	Gentiobiose	+	α-D-Glucose	+	1% Sodium Lactate	+
Starch hydrolysis	−	Sucrose	+	D-Mannose	+	Lincomycin	+
V-P test	+	Turanose	−	D-Fructose	+	Guanidine HCl	+
5% NaCl	+	Stachyose	+	D-Galactose	+	Tetrazolium Blue	+
10% NaCl	+	d-Raffinose	+	3-Methyl-D-Glucose	−	Tetrazolium Violet	+
Gelatin liquefaction test	+	α-d-Lactose	○	L-Fucose	○	Nalidixic acid	−
Citrate utilization test	+	d-Melibiose	+	L-Rhamnose	+	Vancomycin	+
Urease	−	β-Methyl-d-Glucoside	+	Inosine	+	Lithium Chloride	○

+, positive; − negative; ○, critical.

### Plant Growth Promoting Features

This finding indicated that the strain could fix nitrogen and dissolve phosphorus and potassium. Among the plant growth-promoting (PGP) traits, the test organism produce IAA, GA3, ZA, iron carrier and ACC deaminase (**Table 2**).

**Table 2:** Plant growth-promoting traits of HG-1.

Plant growth promoting properties	Activity
Nitrogen fixation	13.105 ± 0.858 mg N/g glucose
Phosphate solubilizing	74.298 ± 7.236 µg/L
Potassium solubilizing	9.514 ± 1.317 µg/ml
IAA production	21.652 ± 0.925 μg/ml
GA_3_	0.358 ± 0.009 μg/ml
ZA	1.364 ± 0.018 μg/ml
Siderophore production	+
ACC deaminase production	35.047 ± 2.317 μmol/(mg·h)

Data are means ± standard deviation (SD) (n = 3).

The metabolites of the HG-1 strain were obtained through gas chromatography, and volatile organic compounds (VOCs) with a peak area above 0.15% were analyzed. The results revealed the presence of PGP substances, including 2, 3-butanediol, [R-(R*.R*)] - (9.26%) and 1-hexanol (0.15%). Moreover, the HG-1 strain was determined to produce substances, such as phenethyl alcohol (2.19%), 2-undecanone (2.15%), 2-nonanone (1.20%), 2-nonanol (1.04%), 2-tridecanone (0.96%), 2-heptanone (0.38%), phenol (0.33%), and 2-pentadecanone (0.27%), that inhibit the growth of pathogens (**Table 3**).

**Table 3:** Main volatile substances of HG-1 strain and their functions.

Peak Number	VOCs	Function	Peak area %	References
11	2,3-Butanediol, [R-(R*,R*)]-	Promote plant growth	9.26	(Ryu et al., 2003; Farag et al., 2006)
38	Phenylethyl Alcohol	Inhibit microbial growth	2.19	(Mo and Sung, 2007)
45	2-Undecanone	Inhibit microbial growth	2.15	(Yuan et al., 2012; Melkina et al., 2017)
36	2-Nonanone	Inhibit microbial growth	1.20	(Yuan et al., 2012)
37	2-Nonanol	Inhibit microbial growth	1.04	(Abarca et al., 2017)
55	2-Tridecanone	Inhibit microbial growth	0.96	(López-Lara et al., 2018)
23	2-Heptanone	Inhibit microbial growth	0.38	(Melkina et al., 2017)
26	Phenol	Inhibition of fungal growth	0.33	(Yuan et al., 2012)
64	2-Pentadecanone	Inhibit bacteria growth	0.27	(Giorgio et al., 2015)
20	1-Hexanol	Promote plant growth	0.15	(Blom et al., 2011)

### Effect of HG-1 Inoculation on Soil Physicochemical Properties

The physical and chemical properties of soil are closely related to crop growth. After 30 days of planting the wheat, The pH, and K content were significantly lower than original soil (*P* < 0.05). The EC, organic C, Na and Ca content significantly higher than original soil (*P* < 0.05). After 30 days of inoculation with the HG-1 strain, the physical and chemical properties of soil as well as its nutrient contents were significantly altered. The pH (an increase of 2.40%) and EC (an increase of 7.92%) of uninoculated soil were significantly higher than those of inoculated soil (*P* < 0.05). The Olsen P (an increase of 29.39%), available N (an increase of 18.46%), exchangeable K (an increase of 10.96%), and organic C (an increase of 14.19%) were significantly higher in the inoculated soil than those in the uninoculated soil (*P* < 0.05) (**Table 4**).

**Table 4:** Effects of *Enterobacter cloacae* HG-1 on chemical properties of rhizosphere soil.

Treatment	pH	EC(μs/cm^−1^)	Olsen P(mg/kg)	Available N(mg/kg)	Exchangeable K(mg/kg)	Organic C(g/kg)	Na(g/kg)	K(g/kg)	Ca(g/kg)
Original soil	8.62 ± 0.02a	404 ± 8.04c	8.59 ± 0.28b	62.57 ± 1.69b	337.07 ± 6.33b	19.07 ± 0.38c	1.08 ± 0.05b	0.61 ± 0.02a	0.24 ± 0.03c
CK	8.55 ± 0.03b	504 ± 8.04a	8.95 ± 0.47b	68.41 ± 4.59b	347.02 ± 6.13b	22.70 ± 0.31b	2.09 ± 0.04a	0.16 ± 0.01c	0.41 ± 0.03b
HG-1	8.35 ± 0.01c	467 ± 3.56b	11.58 ± 1.16a	81.04 ± 3.90a	385.04 ± 6.94a	25.92 ± 0.58a	2.02 ± 0.05a	0.24 ± 0.02b	0.55 ± 0.02a

Values are means ± SD. Different lowercase letters in the table represent the test results of significance of difference between uninoculated and inoculated HG-1 soil indicators, P < 0.05.

### Effect of HG-1 Inoculation on Plant Growth Under NaCl Stress

The effect of HG-1 inoculation on wheat biomass indices under salt stress was characterized by performing independent sample t-tests (*P* < 0.05). Results revealed that wheat growth under salt stress was significantly increased in plants inoculated with the HG-1 strain compared with control plants. In inoculated plants, the root length increased by 19.15% (*P* < 0.05), shoot length increased by 18.83% (*P* < 0.05), fresh weight (FW) increased by 16.67% (*P* < 0.05), and dry weight (DW) increased by 17.96% (*P* < 0.05) compared with control plants (**Table 5**).

**Table 5:** Effects of *Enterobacter cloacae* HG-1 on wheat biomass, antioxidant and ion absorption.

	Root length(cm)	Shoot length(cm)	FW(g)	DW(g)	Soluble sugar(μg g⁻¹ FW)	Total protein(mg g⁻¹ FW)	Proline(μmol g⁻¹ FW)	MDA(mmol g⁻¹ FW)	Na^+^(mg g⁻¹ DW)	K^+^(mg g⁻¹ DW)	Ca^2+^(mg g⁻¹ DW)
CK	12.74 ± 0.51b	16.25 ± 0.48b	1.44 ± 0.05b	0.167 ± 0.004b	2.88 ± 0.06a	3.35 ± 0.17b	7.24 ± 0.26b	10.97 ± 0.58a	1.75 ± 0.08a	1.45 ± 0.08b	0.73 ± 0.05b
HG-1	15.18 ± 0.45a	19.31 ± 0.43a	1.68 ± 0.03a	0.197 ± 0.008a	3.01 ± 0.08a	4.35 ± 0.16a	8.14 ± 0.21a	7.98 ± 0.21b	1.20 ± 0.11b	1.75 ± 0.09a	1.15 ± 0.10a

Values are means ± SD. The small letters in the table represent the significant difference between the indexes of uninoculated and inoculated HG-1 wheat, P < 0.05.

Factors that affect plant osmotic regulation, such as the total contents of protein, proline, soluble sugar, and malondialdehyde (MDA), were measured to assess the effects of HG-1 inoculation on wheat resistance to salt stress. In inoculated plants, the soluble sugar content of wheat leaves increased by 4.51% (*P* < 0.05), the total protein content increased by 29.85% (*P* < 0.05), and the proline content increased by 12.43% (*P* < 0.05) compared with those in uninoculated plants. We then compared the MDA level in investigated plants because MDA levels reflect the level of lipid oxidative damage induced by salt stress. The MDA levels in inoculated plants decreased by 27.26% (*P* < 0.05) when compared with those in the uninoculated plants (**Table 5**).

To investigate the role of the HG-1 strain in alleviating wheat ion stress, we measured differences in Na^+^, K^+^, and Ca^2+^ concentrations in inoculated and uninoculated wheat seedlings. The ion analysis results revealed a difference in the ion concentrations of inoculated and uninoculated plants. The Na^+^ concentration in inoculated wheat seedlings was significantly lower than that in uninoculated seedlings (31.43%, *P* < 0.05), whereas the concentrations of K^+^ (20.69%) and Ca^2+^ (57.53%) were significantly higher in the inoculated plants than those in the uninoculated plants (*P* < 0.05) (**Table 5**).

### Microbial Diversity Response to HG-1 Inoculation in the Wheat Rhizosphere Soil

**Figure 2:**
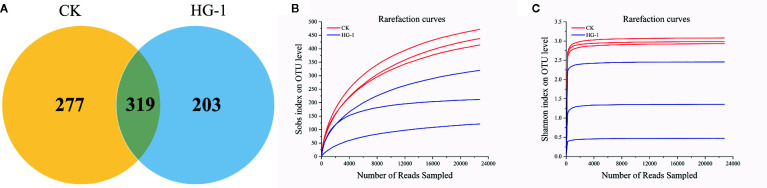
Different treatment samples generate venn diagram, microbial dilution curve and aroma curve based on OUT level. **(A)** venn diagram showing the unique and shared OTUs between different treatments; **(B)** nitrogen-fixing bacteria dilution curve; **(C)** nitrogen-fixing bacteria Shannon-wiener curve.

Miseq sequencing technology was used to sequence the *nifH* gene of the nitrogen-fixing organism, and a total of 261,659 original sequences with an average length of 398.31 bp were identified from six samples. After the screening, 136,578 valid sequences were obtained. A 97% similarity check revealed 799 operational taxonomic units (OTUs), which were obtained using an OTU analysis of nonrepeating sequences. We determined that CK and HG-1 shared 319 OTUs, 277 OTUs were identified in CK alone, and 203 OTUs were identified in HG-1 alone (**Figure 2A**). The microbial diversity index gradually increased with the number of sequencing bars, and the dilution curve became smooth at the last stage, which indicated that sequencing data reached saturation (**Figures 2B, C** **)**, and thus that this study could cover most species of the nitrogen-fixing bacterial community in wheat rhizosphere soil.

The richness, diversity and coverage of NFB communities in wheat rhizosphere soil were analyzed by the diversity index. The Sobs, Chao and Ace indices reflect the richness of the community in the sample. The results in **Figures 2A, B** and **Table 6** showed that compared with the control, the inoculation of HG-1 strain significantly reduced the richness of NFB community in wheat rhizosphere soil (P<0.05) (**Table 6**). Simpson, Shannon index reflects the community diversity in the sample. The results of diversity analysis showed that compared with the control, the inoculation of HG-1 strain did not significantly affect the diversity of NFB community in rhizosphere soil (P<0.05) (**Table 6**).

**Table 6:** Diversity index of nitrogen fixing bacteria in wheat rhizosphere soil samples under different treatments.

Sample	sobs	Shannon	Simpson	ace	chao	coverage
CK	441.33 ± 23.80a	3.00 ± 0.06a	0.18 ± 0.01a	519.82 ± 22.29a	502.69 ± 24.93a	0.9955 ± 0.000170a
HG-1	217.67 ± 81.34b	1.43 ± 0.81a	0.57 ± 0.27a	256.99 ± 99.57b	256.69 ± 100.41b	0.9979 ± 0.001207a

Sobs, chao and ace value are indicators of community richness. Shannon, Simpson and coverage are indicators of community diversity. Values are mean ± SD (n = 3). The small letters in the table represent the significant difference between the indexes of uninoculated and inoculated HG-1 wheat, P < 0.05.

**Figure 3:**
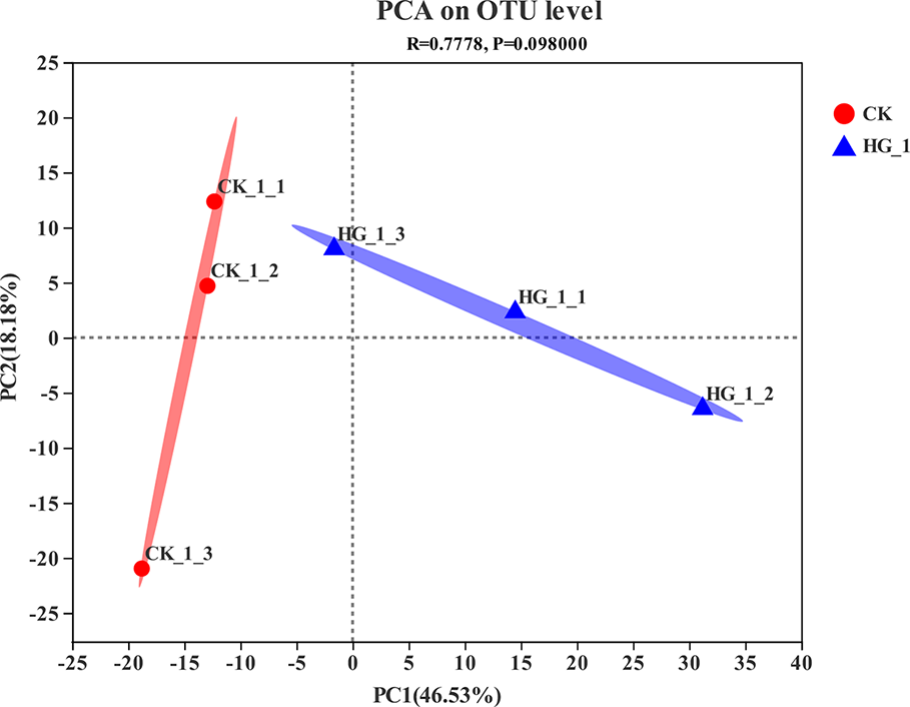
Principal component analysis (PCA) based on Bray-Curtis distances.

The results of PCA showed that the NFB communities in the wheat rhizosphere soil were significantly separated under HG-1 and CK treatment, and ANOSIM analysis further confirmed the significant structural reorganization of the NFB communities in the soil (**Figure 3**).

### Microbial Communities Response to HG-1 Inoculation in the Wheat Rhizosphere Soil

**Figure 4:**
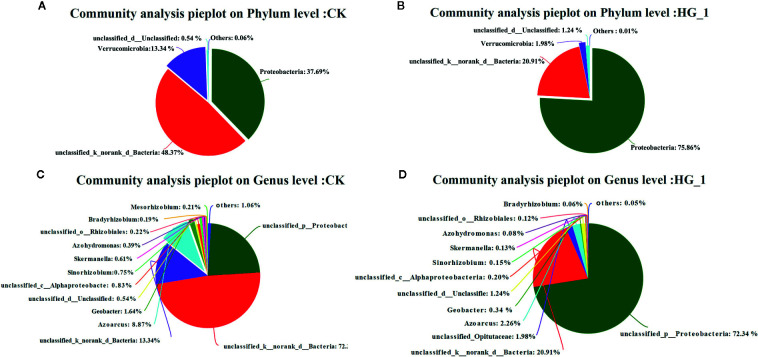
Relative abundance of nitrogen fixing bacteria in wheat rhizosphere soil at phylum and genus level. **(A, B)** relative abundance of nitrogen-fixing bacteria in wheat rhizosphere soil of control group at phylum level, and **(C, D)** relative abundance of nitrogen-fixing bacteria in wheat rhizosphere soil inoculated with HG-1 strain at genus level. Different colors represent different species, and pie area represents the percentage of the species.

**Figure 4** illustrates differences in the composition of NFB communities in inoculated and uninoculated samples at the phylum and genus levels. No differences in the community composition were observed at the phylum level among the investigated samples. However, after inoculation, the abundance of *Proteobacteria* increased from 37.69% to 75.86% and the abundance of *Verrucomicrobia* decreased from 13.34% to 1.98% (**Figures 4A, B** **)**. At the genus level, in the inoculated samples, the abundance of *Geobacter* and *Azoarcus* decreased from 1.64% to 0.34% and from 8.87% to 2.26%, respectively. The R language software vegan package was employed. **Figure 4** illustrates differences identified in each sample for 6 phyla (**Figure 4A**) and 32 genera (**Figure 4B**). The dominant phylum and genus of NFB differed between inoculated and uninoculated plants. The abundance of *Azoarcus*, *Rhodopseudomonas*, *Bradyrhizobium*, *Cyanothece*, *Sinorhizobium*, *Azotobacter*, *Anaeromyxobacter*, and *Vibrio* was lower in inoculated soil than in CK samples, whereas *Azospirillum*, *Anabaena*, and five other genera were only recorded in CK samples.

**Figure 5:**
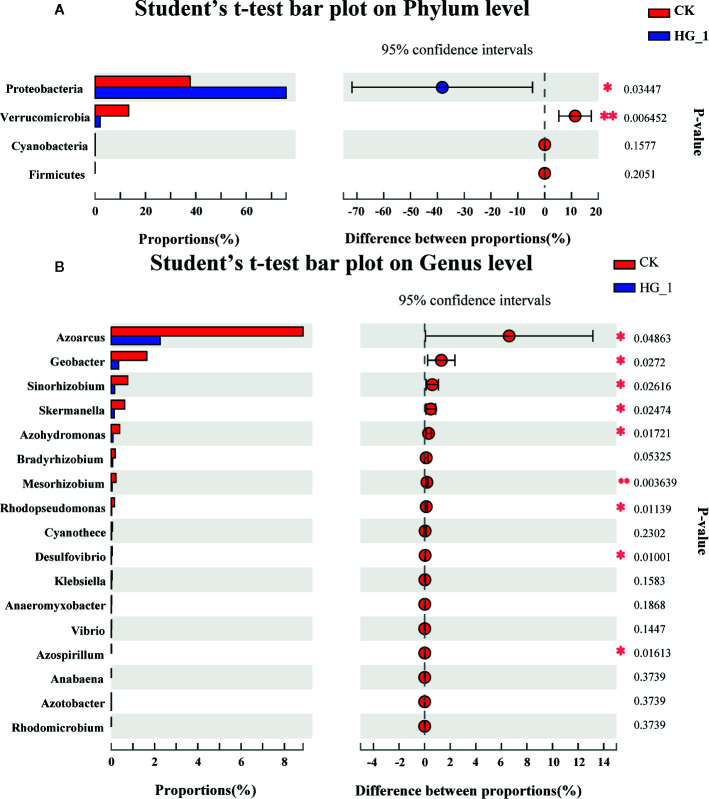
Difference test on **(A)** phylum and **(B)** genus level of nitrogen-fixing bacteria in wheat rhizosphere soil under different treatments. The vertical axis represents the species names at a certain level of classification, and each column corresponding to the species represents the average relative abundance of the species in various groups, with different colors representing different groups. The intermediate region is the difference of the percentage of species abundance between the two groups within the confidence interval set. The color of the dot is the group color with a relatively large proportion of species abundance. The I-type interval on the dot is the upper and lower limit of the difference. Error bars indicate standard errors (n=3). The rightmost is *P* value, *0.01 < *P* ≤ 0.05, **0.001 < *P* ≤ 0.01.

Results revealed that the average relative abundance of Proteobacteria in the inoculated samples increased significantly (*P* ≤ 0.05), and the average relative abundance of Verrucomiorobia was significantly decreased (*P* ≤ 0.01) compared with that in CK samples (**Figure 5A**). Moreover, the differences in the abundance of 27 common bacterial genera were compared. The mean relative abundance of *Azoarcus*, *Geobacter*, *Sinorhizobium*, *Skermanella*, *Azohydromonas*, and *Mesorhizobium* was significantly lower in inoculated samples than in CK samples (*P* ≤ 0.05) (**Figure 5B**).

## Discussion

PGPR can induce plant systemic tolerance (IST) by increasing the concentrations of dissolved phosphorus, ACC deaminase activity, volatile substances, iron carriers, and plant hormones (Farag et al., 2013; Kumari S. et al., 2015). Some studies have shown the potential of *E. cloacae* to be a plant growth promoter and its characteristic of salt tolerant (Bhise et al., 2016; Macedo-Raygoza et al., 2019). In the present study, ten strains were isolated from alkali-saline soil samples and screened to obtain *E. cloacae* HG-1 that had high salt tolerance. The physiological and biochemical and PGP characteristics were measured. We determined that the strain was resistant to antibiotics, including rifamycin SV, troleandomycin, 1% sodium lactate, and lincomycin. Furthermore, the analysis revealed that the strain had characteristics related to processes of nitrogen fixation and the dissolving of phosphorus and potassium and could produce IAA, GA_3_, ZA, iron carriers, and ACC deaminases. Therefore, the strain HG-1 was determined to have the potential to promote plant growth. *E. cloacae* has high nitrogen fixation activity. Numerous studies have shown that *E. cloacae* seems to be not virulent. Based on analyses of *in vitro* hemolysis of red blood cells and antibiotic resistance, *E. cloacae* has no hemolytic activity. At the same time, this strain not only exists widely in rhizosphere soil, but also can be detected and isolated in plants (Hinton and Bacon, 1995; Wang et al., 2012; Macedo-Raygoza et al., 2019). Under both biotic and abiotic stress, it has a growth promotion effect on cowpea, wheat, pea, citrus, corn, banana and other crops and thus is considered a promoter for plant growth (Hinton and Bacon, 1995; Araújo et al., 2002; Kumaran et al., 2010; Ramesh et al., 2014; Khalifa et al., 2016; Macedo-Raygoza et al., 2019). Moreover, *Enterobacter* strains were determined to have various other PGP properties. For example, Liu isolated 11 strains of nitrogen-fixing bacteria from the rhizosphere of the salt-tolerant plant sunflower (*Helianthus tuberosus*) in the Yellow River area, China, and tested the strains for nitrogen-fixing, phosphorus-dissolving, and IAA-producing activities. Among them, *Enterobacter* sp.*N10* can significantly increase the root length and plant height of sunflower and wheat, and is the best rhizobia to increase wheat yield (Liu et al., 2017). Also Li et al., have shown that among the 8 strains of salt-tolerant bacteria, *E. cloacae* HSNJ4 can promote the germination of rape seeds, balance the relative content of IAA and ethylene in seedlings, increase root length, stem length, lateral root content and chlorophyll content, and improve its salt tolerance (Li et al., 2017). *E. cloacae* MDSR9, isolated from soybean rhizosphere, was reportedly capable of producing IAA, iron carriers, ammonia, ACC deaminase, soluble phosphorus, potassium, and zinc. Wheat inoculated with this strain displayed an increase in seedling and seed weight of 39.13% and 49.14%, respectively (Ramesh et al., 2014). Furthermore, after inoculation with *Enterobacter*, the expression of salt stress response genes related to proline biosynthesis in *Arabidopsis* was upregulated (Kim et al., 2014).

The HG-1 strain can improve the physical and chemical properties of saline soil. PGPR are principal biological factors controlling plant growth through the adsorption and desorption of ions and the transformation of nutrients and their availability to crops (Schloss and Jo, 2006). Although the present study is only a preliminary analysis of the effect of PGPR on the rhizosphere soil, numerous studies have determined that the application of microbial agents is also an effective method to significantly reducing soil pH and salinity, accelerate the management of saline-alkali soil, and improve the survival rate of plants in saline-alkali soil (Mbarki et al., 2017). This type of biological management method has a quick effect on the plant growth and is sustainable, low-cost, and does not generate any pollution. Therefore, for the use of microbial agents has great potential to grow plants in saline-alkali soil (Dodd and Pérez-Alfocea, 2012; Paul and Lade, 2014; Nabti et al., 2015; Shrivastava and Kumar, 2015). A high concentration of salt in soil can inhibit plant growth because large numbers of sodium ions affect the nutrient utilization rate of plants and inhibit the activity of various enzymes. Moreover, salt stress affects plant growth by disrupting water balance, causing oxidative stress and ethylene production (Nakbanpote et al., 2014). In this study, soil pH and EC were significantly decreased after the inoculation of soil with HG-1, whereas the available phosphorus, available N, exchangeable K, and organic C in soil increased, which improved the nutritional environment of wheat and reduced the effects of salt stress. The improvement of the soil environment may also increase the adaptability and activity of microorganisms. Biological regulation is an effective measure to improve soil quality and crop yield by promoting the circulation and transformation of soil nutrients and enhancing plants’ absorption of nutrients (Mayak et al., 2004; Egamberdieva et al., 2017). Inoculating plants with these microorganisms can improve soil micro-ecological environments (Lerner et al., 2006; Fließbach et al., 2009).

The HG-1 strain can reduce ion toxicity, osmotic stress, and oxidative damage to plants under salt stress. A study found that *E. cloacae* ZNP-3 can produced ACC deaminase along with several other properties namely IAA production, mineral phosphate solubilization, hydrogen cyanide (HCN) and ammonia production. Its inoculation to wheat plant resulted in a considerable increase in growth parameters, biomass, and chlorophyll content under salinity stress. The inoculation also decreased the accumulation of Na^+^ and increased K^+^ uptake in shoots and roots, leading to maintenance of favorable K^+^/Na^+^ ratios in bacterial-treated plants for alleviating the toxic effect of salt stress (Singh et al., 2017). IAA plays a crucial role in the differentiation of plant cells, tissues, and new organs. The IAA concentration in crops was significantly reduced after salt stress; for instance, the IAA concentration was decreased by approximately 75% in tomato plants (Dunlap and Binzel, 1996). Furthermore, when the isolates of salt-tolerant IAA strains *B. endophyticus*, *B. tequilensis*, and *Planococcus* were re-inoculated into salt-tolerant grass, the germination rate of grass increased by 7%–11%, the shoot length increased by 13%–22%, the root length increased by 44%–57%, and the FW increased by 21%–54% compared with uninoculated plants (Zhao et al., 2016). Gibberellin (GA) is involved in several stages of plant growth and development, including seed germination, leaf growth, photolithogenesis, shoot elongation, flower organ development, and fruit maturation. GA also plays an active role in cell division and elongation and in the regulation of hypocotyl, root, and leaf meristem size (Sun and Gubler, 2004; Daviã¨Re and Achard, 2013; Wang G.L. et al., 2015). GA_3_ is a bioactive GA, which can significantly improve the germination rate of seeds and the water utilization rate of plants under low salt stress (Maggio et al., 2010), as well as can significantly improve the growth condition of plants grown under high salt stress (Javid et al., 2011). The inoculation of rice with *Bacillus amyloliquefaciens*, which produces GA_3_, can increase the salicylic acid concentration in rice, upregulate the expression of endogenous GA-related genes, and significantly promote plant growth (Shahzad et al., 2016). GA produced by *Bacillus* sp. and *Azospirillum* sp. was determined to increase plant nitrogen uptake (Kucey, 2010). CKs mainly regulate plant cell division, apical dominance, chloroplast biogenesis, nutrient regulation, leaf senescence, tube tissue differentiation, light signal conduction development, bud differentiation, and anthocyanin production. CKs also participate in the formation of plant resistance to biotic and abiotic stress (Hwang and Sheen, 2001; O’brien and Eva, 2013). For instance, when *Platycladus orientalis* was inoculated with *B. subtilis*, thereby producing CKs, the CK concentration in the bud increased and the resistance to osmotic stress was stronger in inoculated plants than in uninoculated plants (Liu et al., 2013). In this study, the HG-1 strain produced the plant hormones IAA, GA_3_, and CKs. Our results indicated that the investigated strain could promote the growth and development of wheat seedlings; improve the accumulation of wheat biomass; and positively promote plant height, root length, FW, and DW.

Potassium ions, one of the three main elements in crop nutrition, are ubiquitous in crops. They are involved in almost all physiological and biochemical processes of plants and extensively affect the growth and metabolism of crops. Potassium and sodium have competing regulatory actions on osmotic potential (Ashraf et al., 2005). K^+^/Na^+^ in plants is a valuable indicator of their salt tolerance. Studies have indicated that K^+^ can enhance the salt tolerance of crops (Ruppel et al., 2013). As essential mineral elements, calcium ions are crucial regulators of plant growth and development and are vital components of the plant cell wall (Hepler, 2005). Calcium ions are a protective permeable substance in the vacuole that maintain cell membrane stability and intracellular ion balance. Maintaining the balance of calcium ions in plant cells is crucial in the normal growth of plants (Al-Whaibi et al., 2010; Dayod et al., 2010; Dodd et al., 2010; Gilliham et al., 2011). Proline may play a role in osmoregulation as a crucial component of antioxidant defense during water scarcity, which protects macromolecules and participates in the pentose phosphate pathway (Hare and Cress, 1997; Miller et al., 2010). To date, no accurate conclusion has been drawn regarding the role of PGPR in plant nutrient absorption, transport, accumulation, and calcium ion removal under salt stress. Therefore, studying the mechanisms of K^+^, Ca^2+^, and proline under salt stress from multiple perspectives can help improve plant growth and development. In this study, the inoculation of wheat with the HG-1 strain significantly reduced the accumulation of Na^+^ in the leaves, which might have reduced Na^+^ toxicity in wheat and increased K^+^ and Ca^2+^, thereby maintaining the ion balance. Furthermore, a significant increase in the proline content was detected, indicating that inoculation with HG-1 enhanced the osmotic regulation ability of wheat. Furthermore, K^+^ and Ca^2+^ in wheat leaves were significantly higher in inoculated plants than in uninoculated (control) plants (*P* < 0.05), whereas Na^+^ accumulation was significantly lower (*P* < 0.05). These factors are critical for preserving the function of biological macromolecules, preventing enzyme inactivation, maintaining physiological activity such as photosynthesis, and increasing the tolerance of crops to salt stress (Furusho et al., 2005).

High salinity also leads to increased production of reactive oxygen species (ROS), which can damage the integrity of plant cell membrane systems and affect fatty acids, amino acids, pigments, and other biological molecules in plants (Miller et al., 2010). The MDA level can be used to characterize the degree of oxidative damage caused by ROS (Jain et al., 2001). In this study, the MDA level was significantly lower in plants inoculated with HG-1 than in uninoculated plants, indicating that inoculation with the HG-1 strain could reduce the oxidation of membrane lipids, proteins, and DNA, thereby reducing oxidative damage in plants.

Excessive ethylene production under salt stress can inhibit root growth, and higher ethylene levels in the nodules can reduce the quantity of fixed N (Ma et al., 2002). Reducing stress-induced increases in ethylene levels is beneficial to plant growth (Glick, 2004). Producing ACC deaminase and catalyzing the conversion of ACC (a precursor in ethylene biosynthesis) into ammonia and alpha-ketobutyrate are crucial functions of PGPR, which reduce ethylene levels in plants under salt stress (Senthilkumar et al., 2009; Etesami and Beattie, 2017). Penrose and Glick (2003) reported that ACC deaminase activity was higher than 20 nmol α-KB mg^−1^ protein h^−1^, which could improve the salt tolerance of plants. The ACC deaminase activity of HG-1 was up to 35.047 ± 2.317 μmol α-KB mg^−1^, which indicated that HG-1 could improve the salt tolerance of plants.

VOCs have relatively low molecular weight and are hydrophobic and volatile at room temperature and pressure, which allows them to be readily dispersed in the atmosphere and soil (Hung et al., 2015). Several studies have investigated the volatile metabolites of specific strains. Salme et al. (2014) proposed an effective new method to test VOC strains to promote plant growth and improve plant stress tolerance. Improving our understanding of the metabolites of bacterial strains could solve the problem of food security caused by climate change. For example, *Alcaligenes faecalis* JBCS1294 can reprogram auxin and GA to increase plant salt tolerance by producing adipic acid, butyric acid, and other volatiles (Bhattacharyya et al., 2015). In the present study, the VOCs of salt-resistant *E. cloacae* HG-1 were detected. We determined that various substances could inhibit the growth of pathogenic bacteria, common fungi, and bacteria caused by the soil-borne disease control effect. However, multiple VOCs exhibit synergistic effects, and determining components involved in IST is difficult before isolating each volatile compound for analysis.

The expression “microbiome” can describe the complex and dynamic genetic content of a microorganism living in a specific habitat (Bulgarelli et al., 2013; Reinhold et al., 2015). The role of the rhizosphere soil microbial community in maintaining plant health and improving plant adaptability is being increasingly recognized as the knowledge of this field increases. The related microbial community is considered the second genome of plants (Berendsen et al., 2012; Turner et al., 2013). Therefore, the study of the microbial community structure and diversity in rhizosphere soil is valuable in understanding the mechanism of PGPR and in evaluating the optimal measures for increasing plant salt tolerance. The diversity of soil microbial community is closely related to crop growth and the prevention and control of soil-borne diseases (Miransari, 2013; Sui et al., 2019).

The NFB community in crop rhizosphere soil is a functional bacterial community, which has a critical effect on plant growth (Xun et al., 2019). The number of studies on the characteristics and changes in the nitrogen-fixing microbial community structure in the rhizosphere of major food crops, especially under salt stress, remains insufficient. The HG-1 strain investigated in this study was determined to have high nitrogen fixation activity. The results of this study may provide references for future screening, evaluation, and use of NFB. Evidence indicates that plant roots can rapidly select specific microorganisms and maintain relatively stable communities during any cycle of plant growth, suggesting that the structural basis of the rhizosphere microbial community in the early stages of plant growth is essential (Edwards et al., 2015; Reinhold et al., 2015). Studies have indicated that plants may benefit from the synergy with various interacting microbial communities rather than from the individual members of the community. The results of the present study provide new insights into the future use of culturable beneficial microbiota in enhancing plant salt tolerance and improving agricultural production under saline-alkali conditions. In this type of symbiosis, plants maintain and protect microorganisms using rhizosphere metabolites (Jones et al., 2009), provide carbon sources for their growth, and influence the activity and composition of microbial communities (Mendes et al., 2013). This microbiology-based plant biotechnological research has proven to be more effective than plant breeding and genetic modification methods (Smith, 2014). With the increasing use of microbial fertilizers, the effect of microbial agents on the community structure of nitrogen-fixing microorganisms in the rhizosphere soil of crops has considerable research value and could be used as a new standard for evaluating the effects of soil improvement on plant growth promotion.

Nitrogen is closely correlated with the community structure of nitrogen-fixing microorganisms in the soil. The form and content of nitrogen have significant effects on the composition and diversity of the soil bacterial community (Enwall et al., 2007; Campbell et al., 2010). (Li et al., 2014) reported that the diversity and richness of the soil bacterial community decreased with an increase in nitrogen application. (Fierer et al., 2011) determined that nitrogen application had a significant effect on the bacterial composition but no significant effect on bacterial diversity. In this study, we investigated the effects of inoculation with HG-1 strain on wheat rhizosphere microbial diversity and richness. The Sobs, Ace, and Chao indices demonstrated that after the inoculation of roots with HG-1, the community richness of wheat NFB in rhizosphere soil was significantly lower than in the CK group (*P* < 0.05). We determined that the abundance of *Proteobacteria* in the HG-1 sample increased significantly (*P* ≤ 0.05), whereas the abundance of *Verrucomicrobia* decreased significantly (*P* ≤ 0.01) compared with control samples. At the genus level, we determined that the abundances of *Azospirillum*, *Rhodomicrobium*, *Anabaena*, and two other unclassified genera were lower in HG-1 samples than in CK samples. Moreover, the abundance of *Azoarcus*, *Rhodopseudomonas*, *Bradyrhizobium*, *Cyanothece*, *Sinorhizobium*, *Azotobacter*, *Anaeromyxobacter* and *Vibrio* was significantly lower (*P* ≤ 0.05) in HG-1 samples than in CK samples. The relative abundance of some of these strains was low; however, they played a crucial role in the community function (Shi et al., 2016). The phylum *Proteobacteria* includes numerous bacteria responsible for nitrogen fixation (Wen-Ming et al., 2003; Wang et al., 2015a). Increasing the abundance of *Proteobacteria* in the rhizosphere soil may be of positive significance to the growth of plants. This may be because the HG-1 strain increases the nitrogen content in the soil.

Soil types and plant species are principal factors affecting the microbial community structure (Lakshmanan, 2015; Agler et al., 2016). We hypothesized that one of the principal reasons for this is the decrease in soil pH. Reports have indicated that bacterial diversity decreased with a decrease in pH and vice versa (Bartram et al., 2013). Researchers have suggested that the optimal pH for maintaining microbial diversity is 7.5. Other soil factors are also vital for microbial community diversity as well. For example, ammonium ions, nitrates, and soil organic matter provide nitrogen and carbon sources for nitrogen-fixing microorganisms, and differences in their forms directly affect community composition (Zhiyuan et al., 2010; Collavino et al., 2014). Furthermore, inoculation of plants with a single strain under certain conditions could not effectively improve plant growth and salt tolerance under salt stress, which is because of the relatively weak competitiveness and limited colonization efficiency of inoculated strains compared with indigenous soil microbial communities (Qin et al., 2016). The successful colonization of soil with bacterial strains could significantly affect the microbial community structure in the soil (Wang et al., 2018). In this study, the only variable causing the change in the microbial community structure was the inoculation of soil with the HG-1 strain.

Although *Enterobacter cloacae* is widely distributed in soil and other natural environments, the main problem with using this bacterium as a crop inoculant is the high concentration of cells in the preparation and the risk of its treatment. In addition, when these products are applied to crops directly consumed by humans, they may be harmful to health.

## Conclusion

The present study is the first to screen *E. cloacae* HG-1 with high salt tolerance. We determined that the strain was involved in nitrogen fixation, phosphorus dissolution, and potassium dissolution and that it could produce iron carriers, ACC deaminases, and plant hormones. When the HG-1 strain was inoculated into the wheat rhizosphere under salt stress, the antioxidant activity, osmotic adjustment ability, and biomass accumulation of wheat increased significantly compared with uninoculated plants. Soil nutrient accumulation also increased and soil pH and EC decreased significantly compared with uninoculated plants. Furthermore, we detected that inoculation with the HG-1 strain did not affect the species composition of NFB in wheat rhizosphere soil at the phylum level. However, the average relative abundance of *Proteobacteria* was significantly increased, whereas the abundance of *Verrucomiorobia* was significantly decreased compared with uninoculated plants. At the genus level, the species diversity and relative abundance of samples inoculated with the HG-1 strain decreased compared with uninoculated plants. These findings indicate that the HG-1 strain has screening and abundance regulation effects on NFB in rhizosphere soil and is involved in plant–soil–microbial interactions. The structural change in NFB communities in plant rhizosphere soil may play a key role in nutrient cycling.

## Data Availability Statement

The GenBank accession number of HG-1 strain is MN582993. The url is https://www.ncbi.nlm.nih.gov/nuccore/MN582993.1?The%20report%20=%20fasta.

The raw reads of Illumina MiSeq sequencing data were deposited in the NCBI Sequence Read Archive database (accession number: SRR10420106). The url is https://www.ncbi.nlm.nih.gov/search/all/?term=SRR10420106.

## Author Contributions

CJ conceived and designed the experiment. CJ, ZL, LH, XS, CW, YL and performed the experiment. CJ, XS, HL, CL, and QG analyzed the data. CJ wrote the paper. XL guided the research work and revised the manuscript. All authors contributed to the article and approved the submitted version.

## Funding

This work was supported by the Major Science and Technology Innovation Project of Shandong province (2019JZZY020614), Shandong Agricultural Science and Technology Fund (Forestry, Science, and Technology Innovation) (2019LY003-5), and Science and technology innovation and development special project of Linyi city (2019ZDYF013).

## Conflict of Interest

The authors declare that the research was conducted in the absence of any commercial or financial relationships that could be construed as a potential conflict of interest.

## Supplementary Material

Figure S1 Phylogenetic tree showing relationship between HG-1 and other strains. Bootstrap values indicated on nodes.

Figure S2 Analyses of volatile organic compounds produced by HG-1 strain were performed by gas chromatography.

Figure S3 Analyses of plant hormones secreted by HG-1 strain were performed by HPLC. **(A)** gibberellin (GA_3_), **(B)** zeatin (ZT).
