# Supplementary figures and images for: Effects of Enterobacter cloacae HG-1 on the Nitrogen-Fixing Community Structure of Wheat Rhizosphere Soil and on Salt Tolerance
Source: Front Plant Sci. 2020 Jul 17;11:1094. doi: 10.3389/fpls.2020.01094 (PMC7380250; doi:10.3389/fpls.2020.01094)

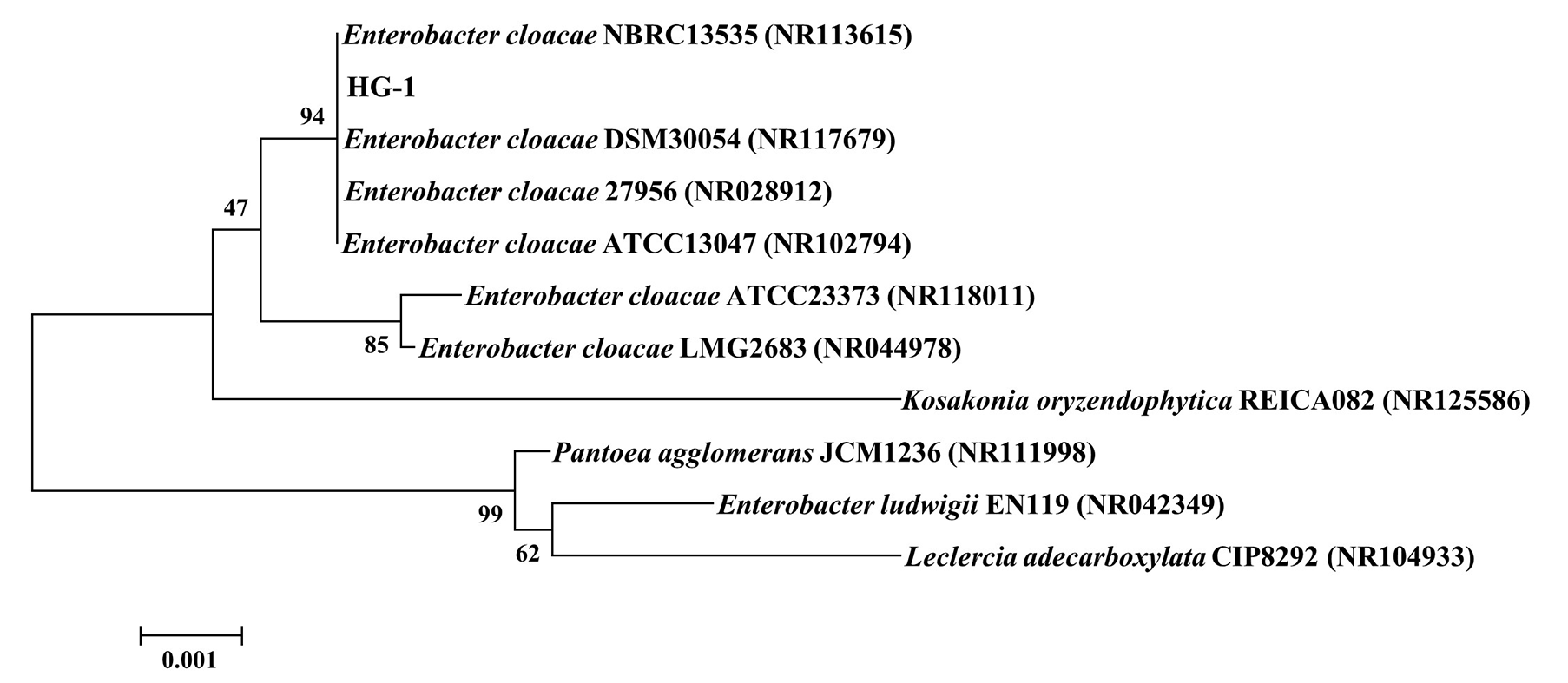

Supplement: Figure S1 — Phylogenetic tree showing relationship between HG-1 and other strains. Bootstrap values indicated on nodes. [file Image_1.tif]

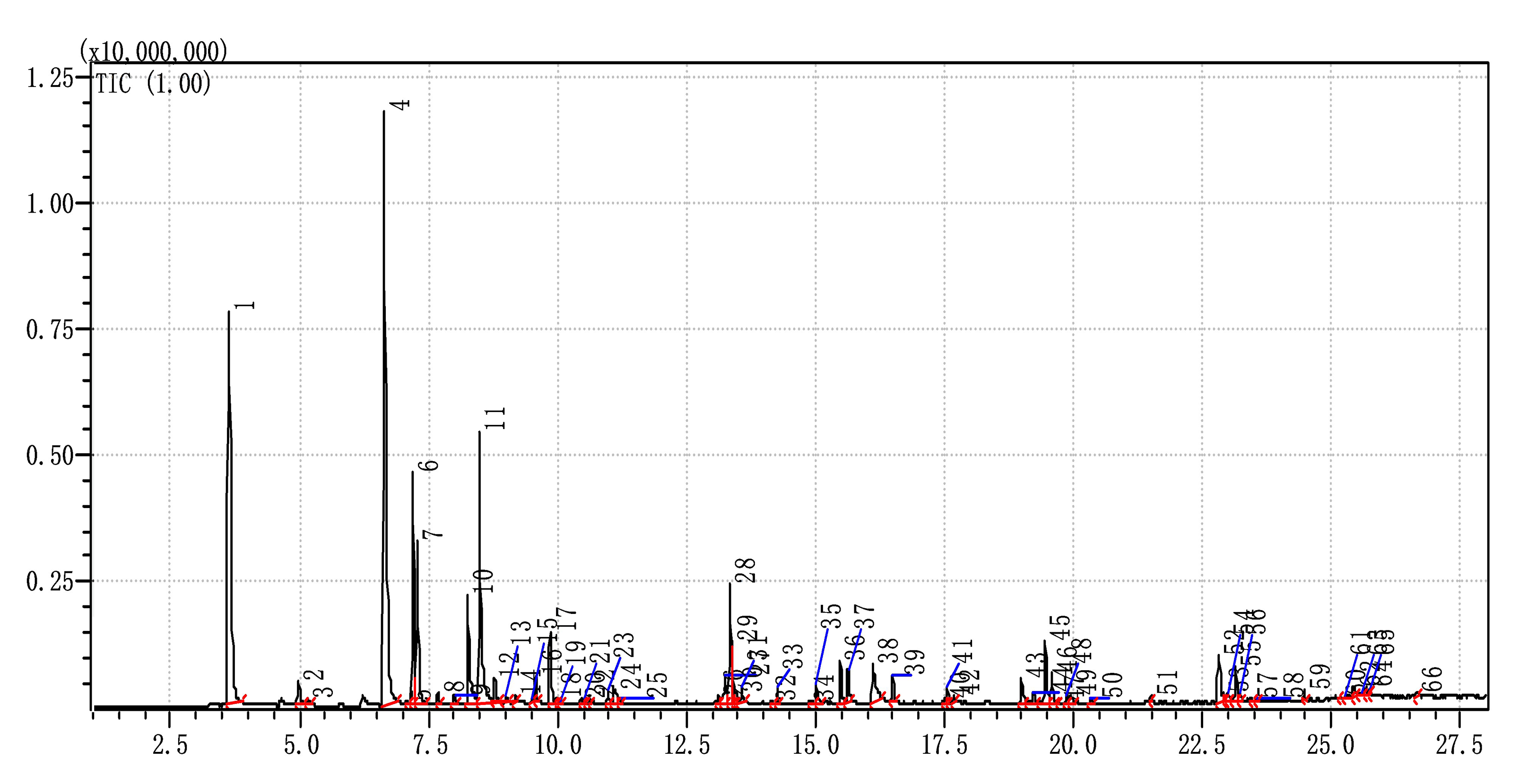

Supplement: Figure S2 — Analyses of volatile organic compounds produced by HG-1 strain were performed by gas chromatography. [file Image_2.tif]

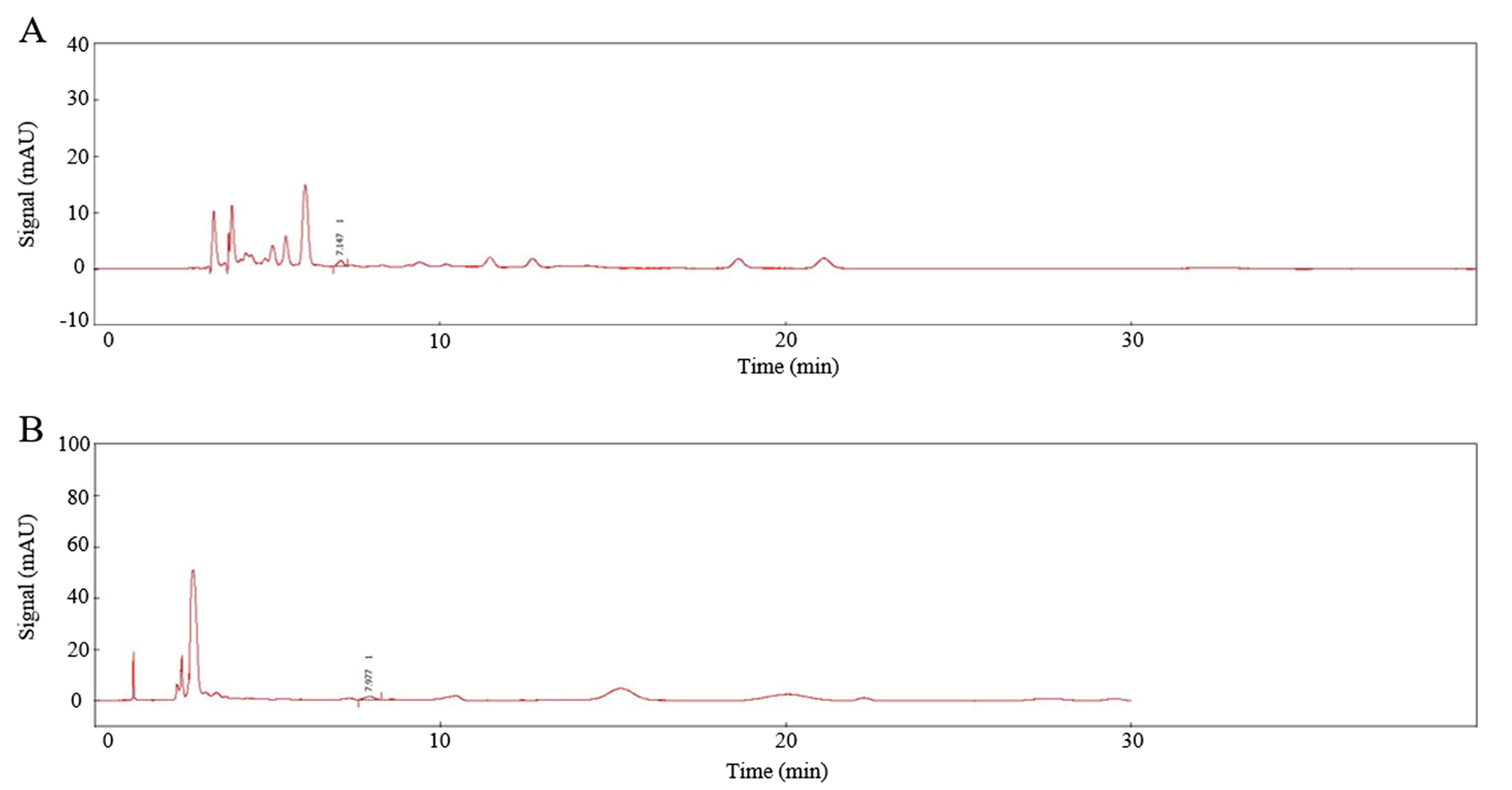

Supplement: Figure S3 — Analyses of plant hormones secreted by HG-1 strain were performed by HPLC. (A) gibberellin (GA3), (B) zeatin (ZT). [file Image_3.tif]
